# Cost-Effectiveness of Colorectal Cancer Genetic Testing

**DOI:** 10.3390/ijerph18168330

**Published:** 2021-08-06

**Authors:** Abdul Rahman Ramdzan, Mohd Rizal Abdul Manaf, Azimatun Noor Aizuddin, Zarina A. Latiff, Keng Wee Teik, Gaik-Siew Ch'ng, Kurubaran Ganasegeran, Syed Mohamed Aljunid

**Affiliations:** 1 Department of Community Health, Faculty of Medicine, Universiti Kebangsaan Malaysia, Jalan Yaacob Latif, Bandar Tun Razak, Cheras, Kuala Lumpur 56000, Malaysia; abdul.rahman@ums.edu.my (A.R.R.); azimatunnoor@ppukm.ukm.edu.my (A.N.A.); 2 Department of Public Health Medicine, University of Malaysia Sabah, Jalan UMS, Kota Kinabalu 88400, Malaysia; 3 Department of Paediatrics, Faculty of Medicine, Universiti Kebangsaan Malaysia, Jalan Yaacob Latif, Bandar Tun Razak, Cheras, Kuala Lumpur 56000, Malaysia; zarinaal@ppukm.ukm.edu.my; 4 Genetic Department, Hospital Kuala Lumpur, Jalan Pahang, Kuala Lumpur 50586, Malaysia; wtkeng@moh.gov.my (K.W.T.); gaiksiew@moh.gov.my (G.-S.C.); 5 Clinical Research Center, Seberang Jaya Hospital, Ministry of Health Malaysia, Penang 13700, Malaysia; 6 Department of Health Policy and Management, Kuwait University, 320 St, Hawally 13110, Kuwait; syed.aljunid@hsc.edu.kw

**Keywords:** colorectal cancer, genetic testing, cost-effectiveness, quality of life, economic evaluation

## Abstract

Colorectal cancer (CRC) remains the second leading cause of cancer-related deaths worldwide. Approximately 3–5% of CRCs are associated with hereditary cancer syndromes. Individuals who harbor germline mutations are at an increased risk of developing early onset CRC, as well as extracolonic tumors. Genetic testing can identify genes that cause these syndromes. Early detection could facilitate the initiation of targeted prevention strategies and surveillance for CRC patients and their families. The aim of this study was to determine the cost-effectiveness of CRC genetic testing. We utilized a cross-sectional design to determine the cost-effectiveness of CRC genetic testing as compared to the usual screening method (iFOBT) from the provider’s perspective. Data on costs and health-related quality of life (HRQoL) of 200 CRC patients from three specialist general hospitals were collected. A mixed-methods approach of activity-based costing, top-down costing, and extracted information from a clinical pathway was used to estimate provider costs. Patients and family members’ HRQoL were measured using the EQ-5D-5L questionnaire. Data from the Malaysian Study on Cancer Survival (MySCan) were used to calculate patient survival. Cost-effectiveness was measured as cost per life-year (LY) and cost per quality-adjusted life-year (QALY). The provider cost for CRC genetic testing was high as compared to that for the current screening method. The current practice for screening is cost-saving as compared to genetic testing. Using a 10-year survival analysis, the estimated number of LYs gained for CRC patients through genetic testing was 0.92 years, and the number of QALYs gained was 1.53 years. The cost per LY gained and cost per QALY gained were calculated. The incremental cost-effectiveness ratio (ICER) showed that genetic testing dominates iFOBT testing. CRC genetic testing is cost-effective and could be considered as routine CRC screening for clinical practice.

## 1. Introduction

Colorectal cancer (CRC) has been reported as the third most commonly diagnosed cancer after lung and breast cancers [1] and the second leading cause of cancer mortality after lung malignancy worldwide [2,3,4]. In Malaysia, approximately 3000 new CRC cases are reported annually [5], constituting the second largest cancer incidence rate after breast malignancy and the third leading cause of cancer deaths. While the annual global incidence and mortality rates of CRCs comprise approximately one million cases and around 600,000 deaths, respectively, the bulk of these diagnosed cases from large surveillance systems or registry data are of hereditary and familial CRCs [6,7,8]. 

As it is a genetically related cancer, genetic testing is one useful tool for screening the risk of cancer inheritance among family members of CRC patients [9,10,11,12,13,14]. Mutations in the MLH1, MSH2, MSH6, and PMS2 genes increase the risk for developing CRCs, especially in Lynch syndrome [15,16,17,18,19,20,21,22].

As the global burden of CRCs is expected to be commensurate with population aging, the costs of cancer treatments are projected to escalate simultaneously [23,24,25,26,27,28,29,30,31,32]. CRC treatment costs and expenditures can be reduced significantly if screening efforts are carried out early, actively, and rigorously [33,34]. Studies in the literature have highlighted that the probability of survival is greater among CRC patients in developed nations due to advancement of screening capacities, detection, and treatment modalities [35,36,37,38].

Although several conventional screening methods are available for CRC detection [39,40,41], there is a need to explore newer interventions with better diagnostic accuracy and performance to conduct early risk assessments for populations susceptible to hereditary CRCs. The current available interventions in Malaysia include the immunological-based fecal occult blood test (iFOBT) and the colonoscopy test for at-risk populations aged 50 years and above [42]. According to the Malaysian Clinical Practice Guidelines for Colorectal Cancer, medium- and high-risk groups are referred to colonoscopy, while CRC genetic testing is optional [43]. Genetic testing in Malaysia is still a less widely used and underutilized screening mode for CRCs [43]. As perceived susceptibility to CRC is attributable to family history in younger age groups [44], it is timely to expand CRC genetic testing capacities as a routine mode for screening in the effort to detect CRCs early, especially in the younger population with a family history of cancer. However, to adopt newer interventions for practice, it is crucial to convince stakeholders and policy makers on the costs and effectiveness of such interventions. The need for effective use of scarce resources has been consistently extended to all aspects of medicine and public health practice. Clinicians and public health advocates have always been concerned with prudent use of resources, and they make careful decisions that take into account both the effectiveness and the costs of the interventions or treatment involved.

Economic evaluation is important to ensure optimal use of the available resources to achieve the desired results. With limited resources in the healthcare system, cost-effectiveness analysis is a useful tool to assess cost–outcome relationships prior to introducing newer, compatible, and more effective interventions. CRC genetic testing is an alternative to the current screening methods for detecting CRC at an early stage. Research on the cost-effectiveness of CRC genetic testing is a key step to obtain accurate and complete information before policy makers decide to expand its use nationally [45].

Studies that conducted cost-effectiveness analysis of CRC genetic testing [46,47,48,49,50,51] concluded that genetic testing is cost-effective in the detection of colorectal malignancy. To the best of our knowledge, there have been no studies of cost-effectiveness related to CRC genetic testing conducted in Malaysia to date. Underlying the premise of healthcare resource scarcity in the quest to maximize health gains for CRC susceptibles and survivors in Malaysia, the aim of this economic evaluation is to determine the cost-effectiveness of CRC genetic testing from the perspective of healthcare providers in Malaysia.

## 2. Materials and Methods

### 2.1. Study Design and Setting 

A cross-sectional study was conducted among CRC patients at three tertiary hospitals in Malaysia from September 2018 until November 2019. 

### 2.2. Study Participants

Study participants were recruited via convenience sample based on the sampling frame of name lists of CRC patients who underwent either iFOBT screening tests or genetic testing at the relevant hospitals. Malaysian adults aged 18 to 85 years of age who were able to read and converse in English or Malay were recruited. Participants deemed physically or mentally unfit to be administered a questionnaire were excluded. Written consents were obtained from those who agreed to participate. A validated questionnaire was used for data collection, consisting of socio-demographic characteristics, health-related quality of life (HRQoL), and resource utilization forms.

### 2.3. Study Perspective, Instruments, and Resources Used

A cost-effectiveness analysis of CRC genetic testing was conducted relative to its comparator, the iFOBT, from the healthcare providers’ perspective in Malaysia, particularly for the Ministry of Health Malaysia as a supplier’s perspective across specialist referral hospitals. Resource utilization forms were used to obtain information related to the costs of the intervention and treatment, while a self-administered questionnaire was used to collect the demographic information of the participants and HRQoL measured as a utility score. 

### 2.4. Outcomes

To determine the effectiveness of a new intervention, the HRQoL of CRC patients was measured using a validated EuroQol instrument, the EQ-5D-5L scale. This tool is often used to determine utility in terms of quality-adjusted life-years (QALYs) during health economic evaluations [52,53]. The Malaysian version of the EQ-5D-5L has been previously validated and adopted for use in the Malaysian healthcare setting [54,55,56]. The EQ-5D-5L valuation for the Malaysian population was used [55].

The EQ-5D-5L is a multi-attribute utility scale (MAUS) that contains a set of questions used to obtain a self-description of current HRQoL across five domains (an EQ-5D descriptive system) and a rating on a visual analog scale (VAS). The five dimensions of the EQ-5D are mobility, self-care, usual activities, pain or discomfort, and anxiety or depression; these are used as a health profile and converted to an index score to describe the utility value for the current health state. For the VAS rating scale, participants are required to rate their health status in a scale of 0–100, in which a higher rating indicates better HRQoL [53]. Participants should mark on the scale to describe their current health status [57].

Life-years (LYs) gained for CRC patients were estimated by multiplying the mean survival time according to the stage of CRC (according to the Malaysia Study on Cancer Survival report) by the number of patients undergoing genetic testing or the iFOBT [58]. Meanwhile, the QALYs for CRC patients were estimated by multiplying the LYs with the mean utility score according to the stage of cancer. The years of life gained by patients who underwent CRC genetic testing were calculated by obtaining the difference between the LYs of patients who underwent genetic testing and the LYs of patients who underwent the iFOBT.

### 2.5. Estimating Resources Used and Cost Analysis

In this study, we adopted single-study-based economic evaluation. The types of costs used in this study were capital and recurrent costs. Capital costs consist of building costs and equipment costs. Recurrent costs consist of human resource costs, administration or overhead costs, utility costs, maintenance costs, medication costs, consumables, and laboratory investigation costs. The cost analysis method adopted in this study was based on those in previous studies [59,60]. The method was based on cost analysis guidelines in primary health care and methods for economic evaluation for healthcare programs [61,62]. All cost data were collected from 1 July 2018 to 28 February 2019 via central costs and resource utilization forms.

Two costing methods were used in this study, namely, the top-down costing method and the activity-based costing method formulated through a clinical pathway. The top-down costing method starts with the total expenditure divided by the total number of patients undergoing genetic testing and the iFOBT, then multiplied by the average number of visits to obtain the average cost per patient per visit. Meanwhile the activity-based costing method is a method that allocates costs to products and services by assigning costs to all activities used directly for undergoing CRC screening. 

### 2.6. Cost-Effectiveness Analysis

Cost-effectiveness analysis was performed based on the costs, utility scores, and mean survival time. The cost-effectiveness in this study is presented as cost per LY gained and cost per QALY computed. It was calculated as cost/effectiveness (LY/QALY). The incremental cost-effectiveness ratio (ICER) was calculated as the cost-effectiveness of genetic testing minus the cost-effectiveness of the iFOBT.

### 2.7. Assumptions and Analytical Choice

In the current study, we adopted cost-effectiveness analysis for diagnostic testing as compared to conventional health economic evaluations that apply such approaches to either new interventions or treatment modalities. Such economic evaluations require the value of diagnostics to be established and integrated with prognostics of health status [63], which explicitly requires the diagnostic accuracy and performance values of new assessments to be synthesized in comparison to conventional tools to warrant an economic evaluation analysis to support policy decisions. In this study, we adopted diagnostic performance values based on a recent systematic review of accuracy in CRC genetic testing [64]. The pooled estimates for sensitivity and specificity were 71% (95% CI 66, 75%) and 95% (95% CI: 93, 97%), respectively. Relative to the accuracy of the iFOBT, the pooled estimated sensitivity was 31% (95% CI: 25, 38%), while the pooled specificity was 87% (95% CI: 86, 89%). These values suggest that genetic testing can detect CRC better at an early stage, promising better health outcomes. The outcome of these performance values forms the basis of the current economic evaluation of genetic testing and justification that it is worthwhile. As a decision tree for an economic model could not be established in view of resource and data scarcity on CRC genetic testing in Malaysia, we required strong assumptions to support the execution of cost-effectiveness analysis of CRC genetic testing. The assumptions made in this study include the following:

- A better screening method is able to detect CRC at an early stage;
- The earlier the stage at which CRC is diagnosed, the better the QALY gain;
- Samples in both screening groups had been tested positive and confirmed to have CRC;
- Total cost management includes the costs of screening and treatment.

### 2.8. Currency, Price Date, and Conversion

All unit costs were adjusted to financial year 2019 using Malaysia consumer price indexes. These costs were then converted into U.S. Dollars using the exchange rate for 2019 (1 USD to 4.14 RM).

### 2.9. Data Analysis

Descriptive and inferential statistics were employed to explore sample characteristics, HRQoL, and the cost of CRC screening methods. The results for continuous variables are presented as means and standard deviations (SDs), and medians. For categorical data, the results are presented as frequencies and relative frequencies (%). Where inferences were made, non-parametric Mann–Whitney U, Kruskal–Wallis, and Chi-Square tests were conducted. Statistical significance was set at *p* < 0.05. Cost analysis and cost-effectiveness analysis for CRC genetic testing was performed. The resulting ICER is graphically presented in a cost-effectiveness (CE) plane.

Variation in costs (mean difference and 95% confidence intervals) yielded through one-sample t-testing is reported to handle stochastic uncertainty. To handle deterministic uncertainty, sensitivity analysis was performed based on the principle of cost reduction. This was done by changing the discount rate from 3% to 5%, in addition to the base case, and was applied to both costs and consequences of the tests. All data were analyzed using SPSS Software version 27.0 (IBM, Armonk, NY, USA). 

## 3. Results

### 3.1. Sample Characteristics

Table 1 shows the sample characteristics. Of the 200 CRC patients recruited, 100 (50.0%) had an iFOBT, while 100 (50.0%) underwent genetic testing. A majority of the CRC patients were men (55%) and aged ≤50 years old (36%). Most patients were Malays (70.0%), married (78.5%), and had a secondary education (58.5%). The bulk of the patients were working (72.5%) with a monthly household income in the range 1500–3500 RM (46.5%). The mean (SD) age of CRC patients was 52.9 (15.8) years. The mean (SD) age of patients who had an iFOBT was 54.9 (15.3) years, while the mean (SD) age of patients who underwent genetic testing was 47.2 (16.2) years. 

### 3.2. Cost Analysis 

The cost for one patient who had an iFOBT or genetic testing in a year was estimated using the top-down and activity-based costing methods. The total cost for one patient who had an iFOBT in a year was USD 372.83, while the total cost for one patient who underwent genetic testing was USD 976.26 (Table 2). 

### 3.3. Outcomes

Table 3 shows the proportions of patient-reported problems among those who underwent the iFOBT or genetic testing in each dimension of the EQ-5D-5L. The problem least reported amongst patients who underwent iFOBT or genetic testing was regarding self-care (29.9% vs. 31.0%, *p* = 0.869). The highest reported problem was pain or discomfort among patients who underwent the iFOBT or genetic testing, and the difference was statistically significant (43.3% vs. 74.1%, *p* < 0.001). The overall mean (SD) utility score for all patients was 0.787 (0.273). The mean (SD) utility score for patients who underwent an iFOBT was 0.801 (0.264), while the mean (SD) utility score for patients who underwent genetic testing was 0.744 (0.296). On the rating scale, the mean (SD) VAS score was 73.58 (18.47) for the overall sample, while the mean (SD) VAS scores for patients who underwent the iFOBT and genetic resting were 73.10 (17.28) and 74.93 (21.29), respectively. There were no statistical differences between patients who underwent the iFOBT and those who underwent genetic testing in terms of utility or VAS score (Table 3). Spearman’s correlation was used to determine the relationship between the utility score and the VAS score. There was a strong positive correlation with statistical significance between the utility score and the VAS score (r = 0.742, *p* < 0.001).

### 3.4. Life-Years (LYs) and Quality-Adjusted Life-Years (QALYs)

The numbers of life-years (LYs) for patients according to cancer stage were 5.21 years for patients who underwent the iFOBT and 6.13 years for patients who underwent genetic testing. The number of quality-adjusted life-years (QALYs) for patients who underwent the iFOBT according to cancer stage was 3.44 years, while the number of QALYs for patients who underwent genetic testing was 4.97 years. The mean survival times for patients who underwent the iFOBT and genetic testing are illustrated in Table 4. 

### 3.5. Life-Years (LYs) and Quality-Adjusted Life-Years (QALYs) Gained by Patients Who Underwent Genetic Testing

The number of years of life gained by patients who underwent CRC genetic testing was calculated by obtaining the difference between the LYs of patients who underwent genetic testing (6.13 years) and the LYs of patients who underwent the iFOBT (5.21 years). The number of LYs gained by CRC patients who underwent genetic testing was 0.92 years. The number of QALYs gained for CRC patients who underwent genetic testing was calculated by obtaining the difference between the QALYs of patients who underwent genetic testing (4.97 years) and the QALYs of patients who underwent the iFOBT (3.44 years). The number of QALYs gained by CRC patients through genetic testing was 1.53 years (Table 5).

### 3.6. Cost-Effectiveness Analysis

Table 5 shows the cost and effectiveness values, i.e., LYs and QALYs gained for the iFOBT and genetic testing in base case analysis. The number of LYs gained by patients who underwent genetic testing was higher (6.13 years) as compared to that by patients who underwent the iFOBT (5.21 years). A gain of approximately 4.97 QALYs was observed for patients who underwent genetic testing, as compared to 3.44 QALYs gained for patients who underwent the iFOBT. The provider cost for patients who underwent genetic testing was USD 976.26, approximately USD 603.43 higher than the provider cost for patients who underwent an iFOBT (USD 372.83).

The cost per LY for patients who underwent genetic testing was USD 159.26, which was USD 87.70 higher than the cost per LY for patients who underwent an iFOBT (USD 71.56). The cost per QALY for patients who underwent genetic testing was USD 196.43, which was USD 88.05 higher than the cost per QALY for patients who underwent the iFOBT (USD 108.38). The cost ratios per LY and per QALY were 2.23 and 1.81, respectively, for patients who underwent genetic testing compared to those who underwent the iFOBT.

The costs and outcomes of intervention with similar objectives would be better when compared at the group level rather than the individual level, especially when the outcomes differ by scales or scores. By grouping patients, the QoL scores of CRC patients with different characteristics were considered. In reality, each health intervention that is under consideration for a policy implementation in society should be carried out as a whole. Table 6 exhibits the QALYs gained and testing costs per 100 patients. Following these results, the incremental cost-effectiveness ratio (ICER) was calculated. The ICER was computed based on the difference in the total cost of management for CRC patients between the two test groups divided by the QALY difference between the two test groups. The cost of managing CRC includes the cost of screening and the cost of treatment. QALYs, on the other hand, are the life-years gained (years of survival) multiplied by the quality-of-life score (QoL) once diagnosed with cancer. The information required is the cost of treatment at each stage of CRC, the number of patients at each stage of CRC in both groups, and the QALYs for each stage of CRC (Table 7).

Based on Table 6, it was found that the number of QALYs decreased as the stage of cancer increased. The percentages of early stage I and II CRCs were 70% for genetic testing and 23% for the iFOBT. This means that CRC is found earlier through genetic testing as compared to the iFOBT. Based on the results from Table 7, it was found that the cost of managing CRC patients rose in line with the increased cancer levels. The cost of genetic testing was more than double the cost of the iFOBT. 

Therefore, the incremental cost-effectiveness ratio (ICER) was calculated as follows:

$$\frac{\mathrm{Total cost managing CRC patients screened by GT}\;(\mathrm{USD})-\mathrm{Total cost managing CRC patients screened by iFOBT}\;(\mathrm{USD})}{\mathrm{Total QALY for patients screened by GT}\;(n=100)-\mathrm{Total QALY for patients screened by iFOBT}\;(n=100)}=\mathrm{ICER}$$

$$\frac{\mathrm{USD}\;600,651.33-\mathrm{USD}\;615,948.66}{496.74-343.78}=\frac{-\mathrm{USD}15,297.33}{152.96}=-\mathrm{USD}100.01\;\left(\mathrm{Genetic}\;\mathrm{testing}\;\mathrm{dominates}\right)$$

**Figure 1:**
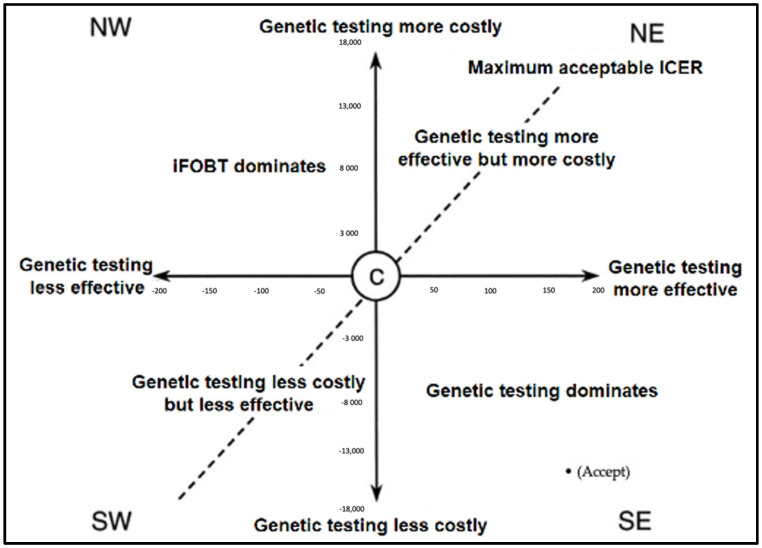
The cost-effectiveness plane and decision rules for CRC genetic testing compared to iFOBT. iFOBT: immunological-based fecal occult blood test; ICER: incremental cost-effectiveness ratio.

Based on the ICER calculations, it was found that genetic testing dominates iFOBT testing when considering the total cost of managing CRC patients and the QALYs gained as the effectiveness. Figure 1 exhibits a cost-effectiveness plane for CRC genetic testing compared to iFOBT testing. The figure shows that the ICER fell in the southeast quadrant, confirming that genetic testing has good value for money (dominant), and decision makers should consider investing in and expanding the use of genetic testing in routine CRC screening for clinical practice.

### 3.7. Sensitivity Analysis

In this study, a one-way sample sensitivity analysis was performed by varying the discount rates from 3% to 5%, in addition to the base case, with the cost and yielded values reported in each sample. The sensitivity analysis results are summarized in Table 8.

## 4. Discussion

The numbers of LYs and QALYs for patients undergoing screening through genetic testing were higher than those for patients undergoing screening through the iFOBT. The cost-effectiveness analysis in this study is presented through cost per LY and cost per QALY. The cost per LY (USD 160.04) for patients undergoing screening through genetic testing was found to be USD 88.48, higher than the cost per LY for patients undergoing screening through the iFOBT (USD 71.56). As for the cost per QALY (USD 217.92), the cost per QALY for patients undergoing screening through genetic testing was USD 126.76, higher than the cost per QALY for patients undergoing screening through the iFOBT (USD 91.16).

The incremental cost-effectiveness ratio (ICER) is the ratio of the increase in cost difference of the two screening methods to the increase in difference of the two effectiveness effects of this study. The lowest incremental cost-effectiveness ratio is the most cost-effective treatment option and is comparable to the value of the Gross Domestic Product (GDP) per capita [65,66]. This ratio is classified into one of three categories: either highly cost-effective, cost-effective, or non-cost-effective. An ICER value of less than one GDP per capita is the most cost-effective choice of CRC screening method, while a value between two and three GDP per capita is considered cost-effective, and a value of more than three GDP per capita is considered ineffective [67,68]. A study by Lim et al. [69] to find the threshold point for Malaysia suggested that there is no single willingness-to-pay (WTP) value for quality-adjusted life-years [69]. The threshold for estimated cost-effectiveness for Malaysia was found to be lower than the threshold value recommended by the World Health Organization.

In this study, the ICER revealed that genetic testing dominates iFOBT testing. Therefore, CRC genetic testing is highly cost-effective when compared to iFOBT testing. These findings coincide with those of a study by Vasen et al. [51], who found that genetic testing was more cost-effective than no testing. Dinh et al. [48] also noted that individual screening for MMR gene mutations, beginning with risk assessment between the ages of 25 and 35 years, followed by genetic testing on individuals whose risk exceeds 5%, is a cost-effective strategy as compared to iFOBT. In fact, cost-effectiveness analytical studies of genetic testing in Taiwan and Australia found genetic testing to be highly cost-effective as compared to no screening [46,47,49,50]. However, the use of genetic testing has been debated because of the increased costs in unnecessary monitoring and treatment that cause it to outweigh the benefits [70,71]. A study by Gallego et al. [72] on the cost-effectiveness of genetic testing for CRC diagnosis among patients referred to cancer genetics clinics showed that the use of hereditary CRC gene panel genetic testing is cost-effective with significant clinical benefits [72]. Most studies used the Markov model to obtain the ICER, but in this study, we compared the costs and results of interventions with similar goals at the group level rather than at the individual level. In reality, every health intervention should be carried out as a whole.

Several studies have used the Markov model to analyze the cost-effectiveness of CRC screening. A study by Wong et al. [73] found that adopting iFOBT annually is a cost-effective screening strategy compared to other recommended screening methods, depending on the willingness to pay for CRC screening among the Chinese population using the Markov model. That study compared the cost-effectiveness of a screening strategy proposed to a Chinese population of 50-year-olds over a 25-year period, namely, annual gFOBT, annual iFOBT, and colonoscopy every 10 years. Data for each screening strategy according to the stage of CRC and cost data for each patient were taken from published studies. Quality-adjusted life-years (QALYs) were measured based on the stage duration and valued based on patient preferences using the SF-6D survey according to cancer stage. The cost-effectiveness result is the additional cost-effectiveness ratio (ICER), i.e., the cost per life-year (LY) and cost per QALY gained. Finally, Wong et al. [73] concluded that iFOBT annually is the most effective and cost-effective CRC screening strategy among the recommended screening strategies, depending on the willingness to pay for screening among the Chinese population [73].

A study in New Zealand using the Markov model also found that CRC screening using the iFOBT every two years was cost-effective. However, the risk of health inequality increased for the Māori population given that their willingness to pay was low [74]. Therefore, to prevent or reduce further health inequalities, attention should be given to disadvantaged population groups when planning and implementing screening programs. A study by Ladabaum et al. [75] also used a model to show that CRC screening initiated at the age of 45 is very cost-effective when compared to no screening or screening initiated at 50 years of age [75]. The use of the iFOBT was found to be more cost-effective, while the use of colonoscopy was more expensive. Although monitoring seemed expensive, it provided significant clinical and economic benefits, especially to those aged ≥ 65 years [76]. The study by Ladabaum et al. (2011) found that genetic testing could reduce the number of deaths from CRC by 7–42% with an ICER of USD 36,200 per LY obtained [77].

Many studies have found that CRC screening modalities are cost-effective. The screening methods considered in these studies were iFOBT (70%), colonoscopy (67%), gFOBT (42%), and sigmoidoscopy (30%), but they were not conclusive on which screening method is the best to adopt for population-based CRC screening programs [78]. While the study by Jahn et al. [79] concluded that the most effective CRC screening method was iFOBT annually or colonoscopy every 10 years, the gFOBT was less effective and more expensive than the iFOBT. Colonoscopy was found to be cost-effective compared to no screening, and an ICER of 15,000 EUR per LY was obtained when colonoscopy was switched with the iFOBT [79]. A cost–benefit analysis study by Heavener et al. [80] also showed that CRC screening gives great returns; however, health systems need to construct payment models to provide better incentives to health providers who implement CRC screening [81]. Other studies have concluded that colonoscopy screening has the potential to become the most cost-effective form of CRC screening [82,83]. However, if the recipient prefers non-invasive testing, other screening strategies, such as genetic testing, could be considered.

### Study Strengths and Limitations

There are some strengths and limitations to this study. In this study, we analyzed the cost-effectiveness of CRC genetic testing in Malaysia. To the best of our knowledge, this analysis is the first to see whether genetic testing is more cost-effective than the iFOBT. Therefore, these findings are able to provide an overview as a base for policy makers to expand the use of genetic testing nationally. We used the universal and frequently used EQ-5D-5L survey form to conduct a health economic assessment of a screening or treatment method. Therefore, the findings of this study can be compared with the results of studies conducted abroad. A study conducted by Huang et al. using EQ-5D-5L found that patients with early stages of CRC had better quality of life as compared to those with late stages of CRC [84]. We found that pain/discomfort was the most frequently reported problem amongst CRC patients. This finding was consistent with previous studies [85,86,87].

The limitations of this study need to be acknowledged. According to Drummond [62], cost analysis is a core element in health economics assessment studies. It needs to be done carefully and preferably from a community perspective. With this, economic evaluation studies will become more extensive and more relevant [81]. We only calculated cost analysis from the provider’s perspective, particularly from that of the Ministry of Health. Because genetic testing has limited availability, being highly selective regarding patients and entirely subsidized by the government of Malaysia, the current study needs to consider the provider’s perspective for economic evaluation of the tests available, and to account for deterministic and stochastic resource use and costs. Appraisal of the economic evaluation in the current study needs to be made with caution, as applying a “societal perspective”, although preferred, will substantially impose a “spillover effect”, as chronic diseases such as cancer at different stages will include caregiver costs and effects, and this does not fundamentally represent the cost burden to the principal provider in Malaysia, the government. 

The study was conducted in three specialist hospitals serving as CRC referral centers. Generalization of the study findings cannot be made. The subjects of this study were only approached by the specialist referral hospitals; thus, the patients being recruited were mostly at an advanced stage of cancer. In Malaysia, the iFOBT and genetic testing are not performed as population-based screening tests for CRC. The iFOBT is only implemented as an opportunistic screening option where individuals attending healthcare facilities are offered the opportunity to undergo screening for CRC, while genetic testing is newly available and offered under limited capacity at a single selected tertiary specialist hospital (Kuala Lumpur Hospital) for high-risk target groups. This situation made patient recruitment to the current study difficult, as only few samples could be recruited during the study period. Being a cross-sectional study, all positive CRC patients at the time of study in those facilities were recruited, and their records were retrospectively examined to determine which test was used. As such, subject recruitment could not be expanded to the population level. Given its acceptable test performance, genetic testing has been adopted in the Malaysian Clinical Practice Guidelines for CRC screening and management; it is offered in selected tertiary specialist hospitals and entirely subsidized by the government of Malaysia. Hence, the provider is highly selective about which patients are assigned to undergo genetic testing. With such methodological, design, and resource implications, this paper is indeed directed, although not in its entirety, towards establishing the value of diagnostics and prognostics for new health technology assessments (HTAs). As cross-sectional studies collect data (utility) at one point of time, CRC patients administered the questionnaire in the current study only responded once, at their particular stage of cancer, thus accruing stochastic uncertainties. Newer technologies differ in the way in which the value is accrued in the user populations, which is critically dependent on the value and availability of downstream healthcare choices [63]. Hence, linked-evidence approaches are the best opportunities for modelling associated with areas of policy, but this study was not powered to execute such modelling analytics; however, it could provide fundamental descriptive insight by incorporating complex information of dichotomous test variables with continuous measures (utilities) or multiple categories (cancer stages) for essential interpretations from the health economic perspective. 

Data completeness is one of the limitations in this study. Secondary data obtained mainly from the Department of Finance and the Department of Medical Records were incomplete for analysis in this study. The lack of fully computerized systems in government hospitals, especially Kuala Lumpur Hospital, resulted in lower data availability. This problem was solved by face-to-face verification with the officers involved, and good explanations were obtained and comparable with trusted sources.

## 5. Conclusions

In conclusion, CRC genetic testing is cost-effective as compared to the iFOBT. This cost-effectiveness analysis of CRC genetic testing provides important and valuable information for the government to consider genetic testing as a screening method to detect cases earlier and for further management to reduce complications, mortality, and economic burden related to CRC. 

## Figures and Tables

**Table 1:** Sample characteristics (*n* = 200).

Characteristics	Total Patients *n* (%)	iFOBT *n* (%)	Genetic Testing *n* (%)	*p*-Value
**Age in years, mean (SD)**	52.9 (15.8)	54.9 (15.3)	47.2 (16.2)	0.159 ^a^
**Age group in years**				0.009 ^b^
≤50	72 (36.0)	30 (30.0)	42 (42.0)	
51–60	49 (24.5)	28 (28.0)	21 (21.0)	
61–70	59 (29.5)	31 (31.0)	28 (28.0)	
≥71	20 (10.0)	11 (11.0)	9 (9.0)	
**Gender**				0.235 ^a^
Men	110 (55.0)	57 (57.0)	53 (53.0)	
Women	90 (45.0)	43 (43.0)	47 (47.0)	
**Ethnicity**				0.113 ^b^
Malay	140 (70.0)	73 (73.0)	67 (67.0)	
Chinese	38 (19.0)	15 (15.0)	23 (23.0)	
Indian	22 (11.0)	12 (12.0)	10 (10.0)	
**Current working status**				<0.001 ^a^
Yes	145 (72.5)	91 (91.0)	54 (54.0)	
No	55 (27.5)	9 (9.0)	46 (46.0)	
Monthly household income (RM) *				0.036 ^b^
<1500	77 (38.5)	41 (41.0)	36 (36.0)	
1500–3500	93 (46.5)	41 (41.0)	52 (52.0)	
>3500	30 (15.0)	18 (18.0)	12 (12.0)	
**Education level**				<0.001 ^b^
No education	17 (8.5)	6 (6.0)	11 (11.0)	
Primary	30 (15.0)	11 (11.0)	19 (19.0)	
Secondary	117 (58.5)	68 (68.0)	49 (49.0)	
Tertiary	36 (18.0)	15 (15.0)	21 (21.0)	
**Marital status**				0.043 ^a^
Single	43 (21.5)	18 (18.0)	25 (25.0)	
Married	157 (78.5)	82 (82.0)	75 (75.0)	
**Insurance coverage**				0.629 ^a^
Yes	28 (14.0)	15 (15.0)	13 (13.0)	
No	172 (86.0)	85 (85.0)	87 (87.0)	
**Family history of cancer**				<0.001 ^a^
Yes	111 (55.5)	40 (40.0)	71 (71.0)	
No	89 (44.5)	60 (60.0)	29 (29.0)	
**Stage of cancer**				<0.001 ^b^
1	16 (8.0)	6 (6.0)	10 (10.0)	
2	77 (38.5)	17 (17.0)	60 (60.0)	
3	79 (39.5)	54 (54.0)	25 (25.5)	
4	28 (14.0)	23 (23.0)	5 (5.0)	
**Treatment**				0.218 ^b^
Surgery	199 (99.5)	100 (100.0)	99 (99.0)	
Radiotherapy	21 (10.5)	8 (8.0)	13 (13.0)	
Chemotherapy	156 (78.0)	87 (87.0)	69 (69.0)	

^a^ Mann–Whitney U test; ^b^ Kruskal–Wallis test; * 1 USD equals RM 4.14 at the time of study; iFOBT: immunological-based fecal occult blood test; SD: standard deviation.

**Table 2:** Cost analysis of the iFOBT and CRC genetic testing (USD).

Type of Cost	Costing Methods	iFOBT Mean (SE)	Genetic Testing Mean (SE)	Difference Mean (95% CI)
**Capital cost (USD)**				
Building	Top-down	62.48 (0.09)	59.93 (0.03)	2.568 (2.565–2.571)
Equipment	Top-down	8.58 (0.01)	8.29 (0.05)	0.300 (0.298–0.303)
**Recurrent cost (USD)**				
Human resource	Activity-based	43.23 (0.48)	56.96 (0.02)	13.789 (13.771–13.808)
Administration/overhead	Top-down	118.13 (0.47)	174.85 (0.10)	56.329 (56.238–56.421)
Utilities	Top-down	69.59 (0.98)	66.75 (0.05)	2.787 (2.770–2.803)
Maintenance	Top-down	4.33 (0.06)	4.15 (0.03)	0.183 (0.180–0.185)
Medication	Activity-based	19.45 (0.66)	0.74 (0.12)	18.751 (18.741–18.760)
Consumables	Activity-based	1.15 (0.01)	0.73 (0.09)	0.407 (0.404–0.409)
Laboratory investigation	Activity-based	45.89 (0.05)	603.86 (0.06)	557.940 (557.931–557.949)
**Total cost (USD)**		372.83 (0.09)	976.26 (0.07)	603.424 (603.422–603.425)

SE: standard error; USD: United States Dollar.

**Table 3:** Proportions of problems reported in the EQ-5D-5L dimensions and the patients’ utility and VAS scores (*n* = 200).

Attribute	Total Patients *n* (%)	iFOBT *n* (%)	Genetic Testing *n* (%)	*p*-Value
Mobility	83 (37.4)	60 (36.6)	23 (39.7)	0.678 ^a^
Self-care	67 (30.2)	49 (29.9)	18 (31.0)	0.869 ^a^
Usual activities	86 (38.7)	67 (40.9)	19 (32.8)	0.277 ^a^
Pain or discomfort	114 (51.4)	71 (43.3)	43 (74.1)	<0.001 ^a^
Anxiety or depression	98 (44.1)	70 (42.7)	28 (48.3)	0.461 ^a^
**Utility score, mean (SD)/median**	0.787 (0.273)/0.861	0.801 (0.264)/0.890	0.744 (0.296)/0.834	0.121 ^b^
**VAS score, mean (SD)/median**	73.58 (18.47)/78.20	73.10 (17.28)/77.50	74.93 (21.59)/80.00	0.288 ^b^

^a^ Chi-square test (χ^2^); ^b^ Mann–Whitney U test.

**Table 4:** Mean survival times for patients who underwent the iFOBT and genetic testing.

Stage	Mean Survival Time	*n*	Total (Years)	Mean Utility Score	QALYs
**iFOBT**					
I	6.71	6	40.26	0.87	35.03
II	6.51	17	110.67	0.74	81.90
III	5.65	54	305.1	0.72	219.67
IV	2.84	23	65.32	0.11	7.19
Total		100	521.35		343.78
Per patient			5.21		3.44
**Genetic testing**					
I	6.71	10	67.1	0.85	57.04
II	6.51	60	390.60	0.82	320.29
III	5.65	25	141.25	0.77	108.76
IV	2.84	5	14.20	0.75	10.65
Total		100	613.15		496.74
Per patient			6.13		4.97

QALYs: Quality-Adjusted Life-Years; iFOBT: immunological-based fecal occult blood test; Stage I, II, III & IV: stage of colorectal cancer.

**Table 5:** Cost-effectiveness analysis of genetic testing.

Item	iFOBT	Genetic Testing
LYs gained	5.21	6.13
QALYs gained	3.44	4.97
Provider cost (USD)	372.83	976.26
Cost per LY (USD)	71.56	159.26
Cost per QALY (USD)	108.38	196.43

iFOBT: immunological-based fecal occult blood test; LYs: Life-Years; QALYs: Quality-Adjusted Life-Years; USD: United States Dollar.

**Table 6:** Quality-adjusted life-years (QALYs) gained.

Stage of Cancer	Life-Years (LY)	iFOBT				Genetic Testing			
		QoL	QALYs	*n*	QALYs (per 100 Patients)	QoL	QALYs	*n*	QALYs (per 100 Patients)
I	6.71	0.87	5.84	6	35.03	0.85	5.70	10	57.04
II	6.51	0.74	4.82	17	81.90	0.82	5.34	60	320.29
III	5.65	0.72	4.07	54	219.67	0.77	4.35	25	108.76
IV	2.84	0.11	0.31	23	7.19	0.75	2.13	5	10.65
Total QALY gain					343.78				496.74
Screening cost (per 100 patients)				USD 37,283.53				USD 97,626.33	

iFOBT: immunological-based fecal occult blood test; LYs: Life-Years; QALYs: Quality-Adjusted Life-Years; USD: United States Dollar.

**Table 7:** Cost of managing colorectal cancer patients.

Stage of Cancer	Treatment [27] Cost (USD)	iFOBT		Genetic Testing	
		*n*	Cost (USD)	*n*	Cost (USD)
I	3,290.34	6	19,742.04	10	32,903.40
II	4,771.01	17	81,107.17	60	286,260.60
III	6031.88	54	325,721.52	25	150797.00
IV	6612.80	23	152,094.40	5	33,064.00
Total treatment cost			578,665.13		503,025.00
Screening cost (per 100 patients)			37,283.53		97,626.33
Total cost of managing CRC patients			615,948.66		600,651.33

iFOBT: immunological-based fecal occult blood test; USD: United States Dollar; CRC: colorectal cancer.

**Table 8:** Sensitivity analysis for the cost-effectiveness of genetic testing versus iFOBT.

Item	Base Case	Discount 3%	Discount 5%
**iFOBT**			
Mean provider cost (USD) (SD)	372.83 (0.06)	361.65 (0.03)	354.19 (0.06)
Life-years (LYs) gained	5.21	5.05	4.95
QALYs gained	3.44	3.34	3.27
**Genetic testing**			
Mean provider cost (USD) (SD)	976.26 (0.05)	946.97 (0.05)	927.44 (0.05)
Life-years (LYs) gained	6.13	5.95	5.82
QALYs gained	4.97	4.82	4.72
Mean provider cost (USD) difference (95% CI)	603.424 (603.422-603.425)	585.318 (585.317–585.319)	573.25 (573.25–573.26)

iFOBT: immunological-based fecal occult blood test; LYs: Life-Years; QALYs: Quality-Adjusted Life-Years; USD: United States Dollar; SD: standard deviation; CI: confidence interval.

## Data Availability

The data presented in this study are available within the article.
